# Supplementary material for: Key priority areas for patient safety improvement strategy in Libya: a protocol for a modified Delphi study
Source: BMJ Open. 2017 Jul 2;7(6):e014770. doi: 10.1136/bmjopen-2016-014770 (PMC5734567; doi:10.1136/bmjopen-2016-014770)
Supplement: Author's manuscript [file bmjopen-2016-014770.draft_revisions.pdf]

BMJ Open

Key Priority Areas for Patient Safety Improvement Strategy in Libya: A Protocol for a modified Delphi Study

Journal:	*BMJ Open*
Manuscript ID	bmjopen-2016-014770
Article Type:	Protocol
Date Submitted by the Author:	17-Oct-2016
Complete List of Authors:	Elmontsri, Mustafa; Imperial College London, Primary Care and Public Health<br>Banarsee, Ricky; Imperial College London, Primary Care and Public Health<br>Majeed, Azeem; Imperial College, Primary Care
**Primary Subject Heading**:	Health services research
Secondary Subject Heading:	Evidence based practice, Health policy, Public health, Global health
Keywords:	Health & safety < HEALTH SERVICES ADMINISTRATION & MANAGEMENT, Risk management < HEALTH SERVICES ADMINISTRATION & MANAGEMENT, Quality in health care < HEALTH SERVICES ADMINISTRATION & MANAGEMENT, Health policy < HEALTH SERVICES ADMINISTRATION & MANAGEMENT, Organisational development < HEALTH SERVICES ADMINISTRATION & MANAGEMENT

SCHOLARONE™
Manuscripts

For peer review only

Title: Key Priority Areas for Patient Safety Improvement Strategy in Libya: A Protocol for a Modified Delphi Study

Mustafa Elmontsri[1BSc (Hons), MSc], Ricky Banarsee[1PhD], Azeem Majeed[1MD FRCP FRCGP FFPH]

Corresponding author: Mustafa Elmontsri, Department of Primary Care and Public Health, School of Public Health, Imperial College London. St Dunstan's Road, 3rd Floor, Reynolds Building, W6 8RP, London. Tel: +44 20 75943362 | Mobile: +447716048560 | Email: m.elmontsri10@imperial.ac.uk

[1]Department of Primary Care and Public Health, School of Public Health, Imperial College London, London, UK

Key words: *Patient safety, adverse events, Developing Countries, Delphi study, System Approach*

Word Count: 2,880

ABSTRACT

Introduction: Patient safety is a global public health problem. Estimates and size of the problem of patient safety in developing countries is scarce. A systems approach is needed for ensuring that patients are protected from hard while receiving care. The primary objective of this study will be to use a consensus-based approach to identify the key priority areas for patient safety improvement in Libya as a developing country.

Design: A modified Delphi study

Methods and Analysis: A three-phase modified Delphi study will be conducted consisting a 3 sequential rounds of anonymous web-based questionnaires. 15 international experts in the field of patient safety will be recruited to priorities areas of patient safety that are vital to developing countries such as Libya. The participants will be given the opportunity to rank a list of elements on 5 criteria. The participants will also be asked to list five barriers that they believe as barriers to implementing patient safety systems. Descriptive statistics will be used to evaluate consensus agreement, including percentage agreement and coefficient of variation. Kendall's coefficient of concordance will be used to evaluate consensus across all participants.

Ethics and dissemination: Ethical approval is currently being sought from Imperial College Research Ethics Committee (ICREC). The findings of the study will be published in a PhD thesis. A manuscript will also be prepared for publication in a high-impact peer-reviewed journal describing the Delphi process and the findings of the study.

For peer review only - http://bmjopen.bmj.com/site/about/guidelines.xhtml

For peer review only

For peer review only - http://bmjopen.bmj.com/site/about/guidelines.xhtml

STRENGTHS AND LIMITATIONS OF THIS STUDY

1. This modified Delphi study is the first study that looks at patient safety research in Libya
2. The Delphi will recruit international experts in the field of patient safety from regional and global organisations to reflect upon their experiences in this field
3. The modified Delphi method will minimise the domination of individual participants through anonymity which will help in eliminating group pressures for conformity
4. Due to lack of patient safety strategy in Libya, this protocol builds upon existing research and data from other developed countries
5. The modified Delphi study aims to include 15 international experts which might limit the generalisability and validity of the findings.

For peer review only

For peer review only - http://bmjopen.bmj.com/site/about/guidelines.xhtml

INTRODUCTION

In the last decade, considerable efforts have been made to improve the safety of healthcare in which it contributed to the widespread acceptance and awareness of the problem of medical harm[1]. In many countries, major progress has been made in assessing the scale and nature of harm[2]. Several studies have examined the extent to which patients are harmed while receiving medical care, for example, the nature and scale of adverse drug events, surgical adverse events, infection and medication prescriptions have been catalogued[1]. Reliable indicators of safety status have been developed over the past decade but the measurement and monitoring of safety continuous to be a challenge[3]. A wide range of contributory factors have been revealed following the analysis of safety incidents. Patient safety incidents in developed countries receive huge attention from the public and the government in which public enquires are ordered to identify the root causes. A recent inquiry in the United Kingdom revealed that the sub-standard performance of individuals due to incapacity or sickness are linked to safety problems in the workplace. However, other causes include "a culture focused on doing the system's business – not that of the patients; too great a degree of tolerance of poor standards and of risk to patients; a failure of communication between the many agencies to share their knowledge of concerns" were contributory factors to poor patient safety performance[4]. In many developed countries, individuals and organisations are being increasingly regulated as well as encouraged to report consistent poor performance to help learn lessons for improvement. The Institute of Medicine report "Crossing the quality chasm: a new health system for the 21st century"[5], indicated that large gaps between the care patients should receive and what they receive exist and recommended the need to search for a new system design to improve performance. Over the past decade, frameworks and models that organise and stimulate the development of theory and practices within the field of patient safety have been proposed. One of the models that encourage a systems approach to patient safety is the System Engineering Initiative for Patient Safety (SEIPS) model[6]. The SEIPS model emphasised upon the need to have an integrated approach to patient safety based on the most well-known model of healthcare quality of Donabedian known as SPO (structure, process and outcomes) model[7]. However, in order for patient safety to be improved, action should be taken at all levels: global, national and local[8]. The global level should focus on sharing knowledge, recommendations and standards across national and local organisations whereas the national level should focus on the development of health system attributes and policies such as the introduction of regulations and coordination of health policies towards improving patient safety. Finally, the local level should execute the interventions proposed to ensure they are relevant and effective.

PATIENT SAFETY IN DEVELOPING COUNTRIES

Countries at all levels of development are affected by patient safety as it is a global public health issue which is also a fundamental requirement in healthcare delivery[9]. Estimates of the size of the patient safety problem in developed countries have been identified by several studies[10,11] whereas in developing and transitional countries such estimates are scarce. The World Health Organization (WHO) estimated that millions of patients suffer disabling injuries or death due to unsafe medical care every year[12]. There is very little known about the scale of unsafe care in developing countries when compared when developed nations. A large-scale study was

For peer review only - http://bmjopen.bmj.com/site/about/guidelines.xhtml

conducted by the WHO Patient Safety arm from 2006 to 2008 to examine the extent of harm in eight countries from the Eastern Mediterranean and Africa in which 26 hospitals took part in the study. 18000 patients experiences were examined in these two regions and the study found that 8 in 100 of the patient studies were exposed to health care-related harmful incident[13]. The study also found that among the factors which contribute to adverse events in developing countries include: inadequate training or supervision of clinical staff, no protocol, no policy or failure to implement, inadequate communication or report and inadequate staffing. There have been infrequent and limited scope of patient safety research in developing and emerging countries[14]. The WHO World Alliance for Patient Safety was created as a result of the alarming issue of patient safety especially in developing countries. The WHO has also requested all member states to "pay the closest possible attention to the problem of patient safety"[15]. It is also recognised by the WHO that patient safety improvement in developing countries require different strategies due the limited capacity and infrastructure of these countries.

AIMS

The aim of this Delphi study is to identify key priority areas for patient safety improvement in developing countries and countries in transition. The study aims to achieve consensus on the most important areas to improve patient safety in developing countries while using Libya as a case-study. The study objectives include the following:

- Identify the most important areas in regards to improving patient safety in developing countries
- To seek expert advice to identify the key factors that are the most important in the improvement of patient safety.
- To identify the barriers that hinder the implementation of patient safety systems
- To develop a patient safety improvement model that is oriented to the Libyan context and capable of improving patient safety in the Libyan healthcare system.

METHODS AND ANALYSIS

A modified Delphi approach will be adopted for this study to identify the key priority areas for patient safety improvement in Libya. The Delphi technique has been widely used in health services and policy research as it supports decision-making and gives a way of structuring a large mass of information and expertise so that informed judgement, forecasting and decision-making can be achieved[16]. It can be used to discuss both numerical and non-quantifiable nature of problems. It is believed that the Delphi technique has the capacity to deal with ambiguity and multi-dimensionality based on its ability to draw on the informed judgement of a group of experts systematically which made it one of the highly reliable tools to support decision-making in the fields of medicine, sociology and policy making[171819]. A key strength of the Delphi technique is the fact that it does not force consensus but helps in identifying where agreement exists among international experts[202122]. Some of the many advantages of the Delphi technique are: ability to conduct a study in geographically dispersed locations as it is the case of this protocol, time and cost effectiveness as well as allows the discussion of complex and broad problems.

USE of Delphi Method in Patient Safety Research

For peer review only - http://bmjopen.bmj.com/site/about/guidelines.xhtml

The Delphi method was used to identify the barriers to Implementation of patient safety systems in healthcare institutions with 23 experts in healthcare quality and healthcare systems approach in which 18 U.S states were represented[23]. The Delphi method was also used to determine developmental progression of quality and safety competencies in nursing education with 18 subject matter experts[24]. It was also used by the WHO World Alliance of Patient Safety to develop an International Classification for Patient Safety in which 253 experts responded to the first round of the Delphi method[20]. The Delphi method was also used to identify and assess for face and content validity of a group of safety indicators with a panel of 30 experts representing all specialities working in labour and delivery units[25]. It was also used by researchers in the U.S to identify priority patient safety outcome measures for use in monitoring and evaluating progress in improving patient safety with 47 national clinical and research experts[26]. The Delphi method was used to identify the causes of patient safety incidents and devise solutions for patient safety in primary care in which 20 physicians and patient safety experts were recurited[21]. A three-round Delphi study was also carried out with a panel of 20 national experts in Canada to develop a set of Canadian consensus-based indicators for the safe use of medication for both in-patient and outpatient settings[27].

Three rounds of the Delphi will take place; each round will be distributed by email. The Delphi method will take place as the following:

- **Pre-Delphi process:** An email Invitation will be sent to the panel of international experts requesting their voluntary participation in the Delphi study. Those who agree to participate will be sent a web-based questionnaire along with a consent form and participant information sheet providing more details about the purpose of the Delphi study.

- **Round 1**: All panel members will be asked to rank a number of elements based on the pre-prepared identified domains and topics using an Online questionnaire. As part of round one, the panel members will be asked to identify five barriers to implementation of patient safety systems in healthcare institutions.

Communication with participants via email will be according to the following schedule:

- Distribution of participant information sheet, consent form and survey link
- Reminder email 1 week later
- Reminder email 2 weeks later.

- **Round 2:** Each panel member will receive feedback of a list of themed topics with supporting statements. Panel members will be asked to rate the importance of each topic using a 5-point Likert scale. They will also be asked to rank the barriers based on the reported common barriers in Round 1.

Communication with participants via email will be according to the following schedule:

- Distribution of the new survey link
- Reminder email 1 week later
- Reminder email 2 weeks later.

For peer review only - http://bmjopen.bmj.com/site/about/guidelines.xhtml

- **Round 3:** Each panel member will receive feedback in the form of a list of themed areas/topics/elements reaching consensus with their supporting statements. Each panel member will be asked to rate each area/topic/element again. Each panel member will be invited to make comments about the prioritisation process.

Communication with participants via email will be according to the following schedule:

- Distribution of the final survey link
- Reminder email 1 week later
- Reminder email 2 weeks later.

Sample and location

Expert panel members will be included from different regions in the world including experts from the United Kingdom, United States, Arab Countries, Africa and from the World Health Organization. The panel members are chosen because of their extensive and invaluable input into the field of patient safety evidenced by their publications in the field. An email will be circulated to each member of the expert panel by the researcher inviting them to participate and respond to the researcher via email to confirm their willingness to participate in the study. Those who wish to participate will be contacted by the researcher with the survey link and a copy of the participant information sheet and consent form. Each questionnaire round will be circulated via email to be completed in the privacy of the individual's home to avoid bias or coercion. All responses within the expert panel will be anonymised prior to collation and circulation.

Analysis

Descriptive statistics will be used to evaluate consensus agreement, including percentage agreement and coefficient of variation. The latter is a normalised measure of dispersion of a probability distribution. The coefficient of variation (CV) is defined as the ratio of the standard deviation to the mean. A level of consensus will also be established. Kendall's coefficient of concordance will be used to evaluate consensus across all participants. This is a non-parametric statistic. It can be used for assessing agreement among the participants. Kendall's W ranges from 0 (no agreement) to 1 (complete agreement).

The Analysis will include:

- Mean rating
- Percentage agreement
- Coefficient of variation
- Kendall's coefficient of concordance

Consensus will be established if:

- Mean rating of 3.5
- Percentage agreement of ≥ 75%
- Coefficient of variation of ≤ 20%
- A median ranking of 4 or more

DISCUSSION

For patient safety to be truly global, and as highlighted by the Institute of Medicine, low and middle-income countries need to be included in the patient safety community[28]. Thus, it could be argued that over the coming 15 years, harm reductions are likely to come from these countries. More importantly, there is a need to ensure that those tools which have been developed in high-income countries can also be developed in low income countries. Countries in transition such as Libya can

For peer review only - http://bmjopen.bmj.com/site/about/guidelines.xhtml

also be fertile for innovation in the healthcare sector. There is also a need for the healthcare system in developing countries to have the right capabilities to produce changes in the delivery and design of services so that the quality and value of healthcare in the 21st century can be improved. Satisfaction, biological and functional parameters should be used to measure the outcomes as part of the re-design efforts of healthcare systems[29]. The capacity of the healthcare system should be built for improvement by successful healthcare leaders of the 21st century in developing countries. Policy makers and all stakeholders in healthcare organisations in developing countries should focus on the need of aligning all organisational strategies for improvement (professional development, financing, operations and education) and should perform patient assessment, data gathering, process assessment and outcome measurement to achieve organisational improvement [30]. In other words, the professional environment, staff, patient and family as well as the leadership and management should all be engaged in all phases of the improvement work. For example, the management and leadership of healthcare organisations can play a significant role in improving patient safety through the establishment of caring relationship with clinical staff, building patient safety infrastructure and promotion of organisational culture change in support the quality and patient safety improvement efforts[31 32].

An agreed set of indicators that are suitable for international comparison which can also help in forming the basis of local measurement systems is highly needed as part of the global movement for patient safety. Global research on patient safety should shift the focus on investigating the issues that face low-income countries and countries in transition in improving their patient safety performance[33]. Research collaboration across countries and international organisations would be very beneficial for countries that have poor infrastructure for conducting empirical research studies. The findings of research could also be disseminated on a global and regional scale to help low-income countries translate these findings into policy recommendations. Patient safety requires a systems approach as it is highly dependent on systemic factors. Redesigning the healthcare system for safer care delivery requires a systems approach in which individual and organisational factors are taken into account[34]. Healthcare institutions would be vulnerable to mistakes in patient safety if a narrow focus on one isolated process without taking into consideration the systematic nature of medical errors is adopted[35]. Hence, this modified Delphi study will help in establishing a point of reference for policy makers in developing countries on the vital elements and components that are required when developing a national strategy for patient safety improvement.

ETHICS AND DISSEMINATION

Ethical approval is currently being sought from Imperial College Research Ethics Committee (ICREC). Confidentiality and anonymity will be kept throughout the study phases and all individual responses will be anonymised prior to collation by the researchers involved in the analysis of this study. Only members of the research team will have access to the codes that will be used for coding the Delphi questionnaires. Data which will be published will not identify the identities or organisations of the individuals participated in the study. The findings of the study will be published in a PhD thesis. A manuscript will also be prepared for publication in a high-impact peer-reviewed journal describing the Delphi process and the findings of the study. We also expect that the findings of this study will be

For peer review only - http://bmjopen.bmj.com/site/about/guidelines.xhtml

disseminated to national, international and regional audiences via conference presentations, posters and seminars. Participants in the Delphi study will also be forwarded a copy of the final report. Finally, it is expected that the findings of this study will initiate collaborative research between countries in developing and developed nations about improving patient safety.

CONCLUSION

All healthcare professionals and stakeholders in the process of healthcare delivery should have personal responsibility to patient safety. A positive culture of patient safety is highly needed in developing countries to ensure that discovering and reporting errors is being rewarded not punished. This modified Delphi study will help in identifying the key priorities for the improvement of patient safety in developing countries using Libya as a case-study and will also help shifting the focus of policy makers in these countries on the key important elements that are needed when developing a national strategy for patient safety improvement. The study will identify the barriers that hinder the implementation of patient safety systems so that comprehensive and efficient national patient safety policies and strategies are developed.

Contributorship Statement

ME and RB prepared the study design and the content of the study protocol. MA further improved the quality of the protocol with important intellectual revisions and assisted in the development of the survey questionnaire for round one. ME is responsible for drafting the protocol manuscript which was then revised and approval by RP and MA.

Competing Interests

None

Funding

This study is part of ME's PhD research which was funded by the Government of Libya. The Department of Primary Care & Public Health at Imperial College London is grateful for support from the NW London NIHR Collaboration for Leadership in Applied Health Research & Care (CLAHRC), the Imperial NIHR Biomedical Research Centre, and the Imperial Centre for Patient Safety and Service Quality (CPSSQ).

Data Sharing Statement

No additional data are available

For peer review only - http://bmjopen.bmj.com/site/about/guidelines.xhtml

REFERENCES

1. Vincent C, Amalberti R. *Safer Healthcare*.; 2016. doi:10.1007/978-3-319-25559-0.
2. Landrigan CP, Parry GJ, Bones CB, Hackbarth AD, Goldmann DA, Sharek PJ. Temporal Trends in Rates of Patient Harm Resulting from Medical Care. *N Engl J Med*. 2010;363(22):2124-2134. doi:10.1056/NEJMsa1004404.
3. Vincent C, Burnett S, Carthey J. Safety measurement and monitoring in healthcare: a framework to guide clinical teams and healthcare organisations in maintaining safety. *BMJ Qual Saf*. 2014;23(April):670-677. doi:10.1136/bmjqs-2013-002757.
4. Francis R. *Report of the Mid Staffordshire NHS Foundation Trust Public Inquiry*. Vol 2013.; 2013. doi:10.1002/yd.20044.
5. Wolfe a. Institute of Medicine Report: Crossing the Quality Chasm: A New Health Care System for the 21st Century. *Policy, Polit Nurs Pract*. 2001;2(3):233-235. doi:10.1177/152715440100200312.
6. Carayon P, Schoofs Hundt A, Karsh B-T, et al. Work system design for patient safety: the SEIPS model. *Qual Saf Health Care*. 2006;15 Suppl 1:i50-8. doi:10.1136/qshc.2005.015842.
7. Donabedian a. The quality of medical care. *Science (80-)*. 1978;200(4344):856-864. doi:10.1126/science.417400.
8. Pronovost PJ, Holzmueller CG, Ennen CS, Fox HE. Overview of progress in patient safety. *Am J Obstet Gynecol*. 2011;204(1):5-10. doi:10.1016/j.ajog.2010.11.001.
9. Vincent CA, Coulter A. Patient safety: what about the patient? *Qual Saf Health Care*. 2002;11(1):76-80. doi:10.1136/qhc.11.1.76.
10. Brennan TA, Leape LL, Laird NM, et al. Incidence of adverse events and negligence in hospitalized patients. Results of the Harvard Medical Practice Study I. *N Engl J Med*. 1991;324(6):370-376. doi:10.1056/NEJM199102073240604.
11. Wilson RM, Runciman WB, Gibberd RW, Harrison BT, Newby L, Hamilton JD. The Quality in Australian Health Care Study. *Med J Aust*. 1995;163(9):458-471. http://eutils.ncbi.nlm.nih.gov/entrez/eutils/elink.fcgi?dbfrom=pubmed&id=7476634&retmode=ref&cmd=prlinks%5Cnpapers3://publication/uuid/82A70B92-AB8B-4AD2-A44F-F45DC132B93A.
12. Schwappach DLB, Conen D. Patient safety - Who cares? *Swiss Med Wkly*. 2012;142(JULY). doi:10.4414/smw.2012.13634.
13. Wilson RM, Michel P, Olsen S, et al. Patient safety in developing countries: retrospective estimation of scale and nature of harm to patients in hospital. *Bmj*. 2012;344(mar13_3):e832. doi:10.1136/bmj.e832.
14. Carpenter KB, Duevel M a, Lee PW, et al. Measures of patient safety in developing and emerging countries: a review of the literature. *Qual Saf Health Care*. 2010;19(1):48-54. doi:10.1136/qshc.2008.031088.
15. Donaldson LJ, Fletcher MG. The WHO World Alliance for Patient Safety: Towards the years of living less dangerously. *Med J Aust*. 2006;184(10 SUPPL.). doi:don10047_fm [pii].
16. Rowe G, Wright G. The Delphi technique: Past, present, and future prospects - Introduction to the special issue. *Technol Forecast Soc Change*.

For peer review only - http://bmjopen.bmj.com/site/about/guidelines.xhtml

2011;78(9):1487-1490. doi:10.1016/j.techfore.2011.09.002.
17. Ziglio E. The Delphi Method and its Contribution to Decision-making. In: *Gazing Into the Oracle: The Delphi Method and Its Application to Social Policy and Public Health*. ; 1996:252.
18. Falzarano M, Pinto Zipp G. Seeking consensus through the use of the Delphi technique in health sciences research. *J Allied Health*. 2013;42(2):99-105.
19. Landeta J. Current validity of the Delphi method in social sciences. *Technol Forecast Soc Change*. 2006;73(5):467-482. doi:10.1016/j.techfore.2005.09.002.
20. Thomson R, Lewalle P, Sherman H, Hibbert P, Runciman W, Castro G. Towards an International Classification for Patient Safety: A Delphi survey. *Int J Qual Heal Care*. 2009;21(1):9-17. doi:10.1093/intqhc/mzn055.
21. Verstappen W, Gaal S, Esmail A, Wensing M. Patient safety improvement programmes for primary care. Review of a Delphi procedure and pilot studies by the LINNEAUS collaboration on patient safety in primary care. *Eur J Gen Pract*. 2015;21 Suppl:50-55. doi:10.3109/13814788.2015.1043725 [doi].
22. Elwyn G, O'Connor A, Stacey D, et al. Developing a quality criteria framework for patient decision aids: online international Delphi consensus process. *BMJ*. 2006;333(7565):417. doi:bmj.38926.629329.AE [pii] 10.1136/bmj.38926.629329.AE.
23. Akins R, Cole BR. Barriers to implementation of patient safety systems in healthcare institutions: leadership and policy implications. *J Patient Saf*. 2005;1(1):9-16. http://journals.lww.com/journalpatientsafety/Abstract/2005/03000/Barriers_to_Implementation_of_Patient_Safety.5.aspx.
24. Barton AJ, Armstrong G, Preheim G, Gelmon SB, Andrus LC. A national Delphi to determine developmental progression of quality and safety competencies in nursing education. *Nurs Outlook*. 2009;57(6):313-322. doi:10.1016/j.outlook.2009.08.003.
25. Haller G, Camparini-Righini N, Kern C, et al. [Patient safety indicators for obstetrics: a Delphi based study]. *J Gynecol Obstet Biol Reprod (Paris)*. 2010;39(5):371-378. doi:10.1016/j.jgyn.2010.05.011.
26. Farley DO, Greenberg MD, Haviland AM, Lovejoy S. Prioritizing Patient Safety Outcomes Measures: Results of an Expert Consensus Process. *Rand Heal*. 2008.
27. Nigam R, Mackinnon NJ, U D, et al. Development of canadian safety indicators for medication use. *Healthc Q*. 2008;11(3 Spec No.):47-53. doi:10.12927/hcq.2008.19649.
28. Zhu H, Das P, Woodhouse R, Kubba H. Improving the quality of tracheostomy care. *Breathe*. 2014;10(4):286-294. doi:10.1183/20734735.011114.
29. Batalden P, Splaine M. What will it take to lead the continual improvement and innovation of health care in the twenty-first century? *Qual Manag Health Care*. 2002;11(1):45-54. doi:10.1097/00019514-200211010-00008.
30. Brandrud AS, Schreiner A, Hjortdahl P, Helljesen GS, Nyen B, Nelson EC. Three success factors for continual improvement in healthcare: an analysis of the reports of improvement team members. *BMJ Qual Saf*. 2011;20:251-259. doi:10.1136/bmjqs.2009.038604.
31. Bradley EH, Holmboe ES, Wang Y, et al. What are hospitals doing to increase beta-blocker use?. *Jt Comm J Qual Saf*. 2003;29(8):409-415. http://ovidsp.ovid.com/ovidweb.cgi?T=JS&PAGE=reference&D=med4&NEWS

For peer review only - http://bmjopen.bmj.com/site/about/guidelines.xhtml

=N&AN=12953605.
32. Künzle B, Kolbe M, Grote G. Ensuring patient safety through effective leadership behaviour: A literature review. *Saf Sci*. 2010;48(1):1-17. doi:10.1016/j.ssci.2009.06.004.
33. Bates DW, Larizgoitia I, Prasopa-Plaizier N, Jha AK. Global priorities for patient safety research. *BMJ*. 2009;338(may):b1775. doi:10.1136/bmj.b1775.
34. Waterson P. A critical review of the systems approach within patient safety research. *Ergonomics*. 2009;52(10):1185-1195. doi:10.1080/00140130903042782.
35. McNutt RA, Abrams R, Arons DC. <McNutt Patient safety efforts should focus on medical errors.pdf>. *JAMA*. 2002;287(15):1997-2001. doi:10.1001/jama.287.15.1997.

For peer review only

Key Priority Areas for Patient Safety Improvement Strategy in Libya: A Protocol for a modified Delphi Study

Journal:	*BMJ Open*
Manuscript ID	bmjopen-2016-014770.R1
Article Type:	Protocol
Date Submitted by the Author:	09-May-2017
Complete List of Authors:	Elmontsri, Mustafa; Imperial College London, Primary Care and Public Health<br>Banarsee, Ricky; Imperial College London, Primary Care and Public Health<br>Majeed, Azeem; Imperial College, Primary Care
**Primary Subject Heading**:	Health services research
Secondary Subject Heading:	Evidence based practice, Health policy, Public health, Global health
Keywords:	Health & safety < HEALTH SERVICES ADMINISTRATION & MANAGEMENT, Risk management < HEALTH SERVICES ADMINISTRATION & MANAGEMENT, Quality in health care < HEALTH SERVICES ADMINISTRATION & MANAGEMENT, Health policy < HEALTH SERVICES ADMINISTRATION & MANAGEMENT, Organisational development < HEALTH SERVICES ADMINISTRATION & MANAGEMENT

For peer review only

For peer review only - http://bmjopen.bmj.com/site/about/guidelines.xhtml

Title: Key Priority Areas for Patient Safety Improvement Strategy in Libya: A Protocol for a Modified Delphi Study

Mustafa Elmontsri[1BSc (Hons), MSc], Ricky Banarsee[1PhD], Azeem Majeed[1MD FRCP FRCGP FFPH]

Corresponding author: Mustafa Elmontsri, Department of Primary Care and Public Health, School of Public Health, Imperial College London. St Dunstan's Road, 3rd Floor, Reynolds Building, W6 8RP, London. Tel: +44 20 75943362 | Mobile: +447716048560 | Email: m.elmontsri10@imperial.ac.uk

[1]Department of Primary Care and Public Health, School of Public Health, Imperial College London, London, UK

Key words: *Patient safety, adverse events, Developing Countries, Delphi study, System Approach*

Word Count: 3,317

ABSTRACT

Introduction: Patient safety is a global public health problem. Estimates and size of the problem of patient safety in developing countries is scarce. A systems approach is needed for ensuring that patients are protected from harm while receiving care. The primary objective of this study will be to use a consensus-based approach to identify the key priority areas for patient safety improvement in Libya as a developing country.

Design: A modified Delphi study

Methods and Analysis: A three-phase modified Delphi study will be conducted using an anonymous web-based questionnaires. 15 international experts in the field of patient safety will be recruited to prioritise areas of patient safety that are vital to developing countries such as Libya. The participants will be given the opportunity to rank a list of elements on 5 criteria. The participants will also be asked to list five barriers that they believe hinder the implementation of patient safety systems. Descriptive statistics will be used to evaluate consensus agreement, including percentage agreement and coefficient of variation. Kendall's coefficient of concordance will be used to evaluate consensus across all participants.

Ethics and dissemination: Ethical approval has been granted from Imperial College Research Ethics Committee (ICREC: 16IC3598). The findings of the study will be published in a PhD thesis. A manuscript will also be prepared for publication in a high-impact peer-reviewed journal describing the Delphi process and the findings of the study.

For peer review only - http://bmjopen.bmj.com/site/about/guidelines.xhtml

STRENGTHS AND LIMITATIONS OF THIS STUDY

1. This modified Delphi study is the first study that focuses on the issue of patient safety in Libya using this methodology.
2. The Delphi will recruit international experts in the field of patient safety from regional and global organisations to reflect upon their experiences in this field
3. The modified Delphi method will minimise the domination of individual participants through anonymity which will help in eliminating group pressures for conformity
4. Due to lack of patient safety strategy in Libya, this protocol builds upon existing research and data from other developed countries
5. The modified Delphi study aims to include 15 international experts which might limit the generalisability and validity of the findings.

For peer review only

INTRODUCTION

In the last decade, considerable efforts have been made to improve the safety of healthcare in which it contributed to the widespread acceptance and awareness of the problem of medical harm[1]. In many countries, major progress has been made in assessing the scale and nature of harm[2]. Several studies have examined the extent to which patients are harmed while receiving medical care, for example, the nature and scale of adverse drug events, surgical adverse events, infection and medication prescriptions have been catalogued[1]. Reliable indicators of safety status have been developed over the past decade but the measurement and monitoring of safety continuous to be a challenge[3]. A wide range of contributory factors have been revealed following the analysis of safety incidents. Patient safety incidents in developed countries receive huge attention from the public and the government in which public enquires are ordered to identify the root causes. A recent inquiry in the United Kingdom revealed that the sub-standard performance of individuals due to incapacity or sickness are linked to patient safety problems in the workplace and led to the occurrence of harm to patients[4]. However, other causes include “a culture focused on doing the system’s business – not that of the patients; too great a degree of tolerance of poor standards and of risk to patients; a failure of communication between the many agencies to share their knowledge of concerns” were contributory factors to poor patient safety performance[4]. In many developed countries, individuals and organisations are being increasingly regulated as well as encouraged to report consistent poor performance to help learn lessons for improvement. The Institute of Medicine report “Crossing the quality chasm: a new health system for the 21st century”[5], indicated that large gaps between the care patients should receive and what they receive exist and recommended the need to search for a new system design to improve performance. Over the past decade, frameworks and models that organise and stimulate the development of theory and practices within the field of patient safety have been proposed. One of the models that encourage a systems approach to patient safety is the System Engineering Initiative for Patient Safety (SEIPS) model[6]. The SEIPS model was constructed based on health systems in developed countries and puts much emphasis upon the role of technology as part of the work system components. The SEIPS model emphasised upon the need to have an integrated approach to patient safety based on the most well-known model of healthcare quality of Donabedian known as SPO (structure, process and outcomes) model[7]. However, in order for patient safety to be improved, action should be taken at all levels: global, national and local[8]. The global level should focus on sharing knowledge, recommendations and standards across national and local organisations whereas the national level should focus on the development of health system attributes and policies such as the introduction of regulations and coordination of health policies towards improving patient safety. Finally, the local level should execute the interventions proposed to ensure they are relevant and effective.

PATIENT SAFETY IN DEVELOPING COUNTRIES

Countries at all levels of development are affected by patient safety as it is a global public health issue which is also a fundamental requirement in healthcare delivery[9]. Estimates of the size of the patient safety problem in developed countries have been identified by several studies[10,11] whereas in developing and transitional countries such estimates are scarce. Among the patient safety risks that are present in developing countries include hospital-acquired infections, surgical complications,

For peer review only - http://bmjopen.bmj.com/site/about/guidelines.xhtml

delays in diagnosis and adverse drug reactions[12]. Similarly, Harrison et al[13] found that medication errors, patient infection and poor maternal and perinatal care are among the common patient safety risks in developing countries in Southeast Asia. The World Health Organization (WHO) estimated that millions of patients suffer disabling injuries or death due to unsafe medical care every year[14]. There is very little known about the scale of unsafe care in developing countries when compared when developed nations. A large-scale study was conducted by the WHO Patient Safety arm from 2006 to 2008 to examine the extent of harm in eight countries from the Eastern Mediterranean and Africa in which 26 hospitals took part in the study. 18000 patients experiences were examined in these two regions and the study found that 8 in 100 of the patient studies were exposed to health care-related harmful incident[12]. The study also found that among the factors which contribute to adverse events in developing countries include: inadequate training or supervision of clinical staff, no protocol, no policy or failure to implement, inadequate communication or report and inadequate staffing. There have been infrequent and limited scope of patient safety research in developing and emerging countries[15]. The WHO World Alliance for Patient Safety was created as a result of the alarming issue of patient safety especially in developing countries. The WHO has also requested all member states to "pay the closest possible attention to the problem of patient safety"[16]. It is also recognised by the WHO that patient safety improvement in developing countries require different strategies due the limited capacity and infrastructure of these countries. Such strategies would need to be developed based on the available resources and expertise while taking into account the lack of regulations and accountability within such health systems.

AIMS

The aim of this Delphi study is to identify key priority areas for patient safety improvement in developing countries and countries in transition. The study aims to achieve consensus on the most important areas to improve patient safety in developing countries while using Libya as a case-study. The study objectives include the following:

- Identify the most important areas in regards to improving patient safety in developing countries
- To seek expert advice to identify the key factors that are the most important in the improvement of patient safety.
- To identify the barriers that hinder the implementation of patient safety systems
- To develop a patient safety improvement model that is oriented to the Libyan context and capable of improving patient safety in the Libyan healthcare system.

Libya is chosen as a case study as the country is undergoing a transitional stage. Libya provides universal health coverage free of charge. The health system in Libya is mainly funded by the state. Health services are delivered through primary health care centres, polyclinics, rehabilitation centres and general hospitals in urban and rural areas.

METHODS AND ANALYSIS

A modified Delphi approach will be adopted for this study to identify the key priority areas for patient safety improvement in Libya. This modified Delphi adopts a structured questionnaire for round 1 and a small sample size[17]. The Delphi technique

For peer review only - http://bmjopen.bmj.com/site/about/guidelines.xhtml

has been widely used in health services and policy research as it supports decision-making and gives a way of structuring a large mass of information and expertise so that informed judgement, forecasting and decision-making can be achieved[18]. It can be used to discuss both numerical and non-quantifiable nature of problems. It is believed that the Delphi technique has the capacity to deal with ambiguity and multi-dimensionality based on its ability to draw on the informed judgement of a group of experts systematically which made it one of the highly reliable tools to support decision-making in the fields of medicine, sociology and policy making[192021]. A key strength of the Delphi technique is the fact that it does not force consensus but helps in identifying where agreement exists among international experts[222324]. Some of the many advantages of the Delphi technique are: ability to conduct a study in geographically dispersed locations as it is the case of this protocol, time and cost effectiveness as well as allows the discussion of complex and broad problems.

USE of Delphi Method in Patient Safety Research

The Delphi method was used to identify the barriers to Implementation of patient safety systems in healthcare institutions with 23 experts in healthcare quality and healthcare systems approach in which 18 U.S states were represented[25]. The Delphi method was also used to determine developmental progression of quality and safety competencies in nursing education with 18 subject matter experts[26]. It was also used by the WHO World Alliance of Patient Safety to develop an International Classification for Patient Safety in which 253 experts responded to the first round of the Delphi method[22]. The Delphi method was also used to identify and assess for face and content validity of a group of safety indicators with a panel of 30 experts representing all specialities working in labour and delivery units[27]. It was also used by researchers in the U.S to identify priority patient safety outcome measures for use in monitoring and evaluating progress in improving patient safety with 47 national clinical and research experts[28]. The Delphi method was used to identify the causes of patient safety incidents and devise solutions for patient safety in primary care in which 20 physicians and patient safety experts were recurited[23]. A three-round Delphi study was also carried out with a panel of 20 national experts in Canada to develop a set of Canadian consensus-based indicators for the safe use of medication for both in-patient and outpatient settings[29].

Three rounds of the Delphi will take place as shown in Figure 1; each round will be distributed by email. The Delphi method will take place as the following:

Insert Figure 1 Here

- **Pre-Delphi process:** An email Invitation will be sent to the panel of international experts requesting their voluntary participation in the Delphi study. The experts come from different geographical areas including those from North Africa and the Middle East, WHO and from the United Kingdom and the United States. The experts are selected based on their familiarity with health systems in developing countries as well as their wealth of publications in the field of patient safety. Those who agree to participate will be sent a web-based questionnaire along with a consent form and participant information sheet providing more details about the purpose of the Delphi study.

For peer review only - http://bmjopen.bmj.com/site/about/guidelines.xhtml

For peer review only

- **Round 1**: All panel members will be asked to rank a number of elements based on the pre-prepared identified domains and topics using an Online questionnaire. As part of round one, the panel members will be asked to identify five barriers to implementation of patient safety systems in healthcare institutions.

Communication with participants via email will be according to the following schedule:

- Distribution of participant information sheet, consent form and survey link
- Reminder email 1 week later
- Reminder email 2 weeks later.

- **Round 2:** Each panel member will receive feedback of a list of themed topics with supporting statements. Panel members will be asked to rate the importance of each topic using a 5-point Likert scale. They will also be asked to rank the barriers based on the reported common barriers in Round 1.

Communication with participants via email will be according to the following schedule:

- Distribution of the new survey link
- Reminder email 1 week later
- Reminder email 2 weeks later.

- **Round 3:** Each panel member will receive feedback in the form of a list of themed areas/topics/elements reaching consensus with their supporting statements. Each panel member will be asked to rate each area/topic/element again. Each panel member will be invited to make comments about the prioritisation process.

Communication with participants via email will be according to the following schedule:

- Distribution of the final survey link
- Reminder email 1 week later
- Reminder email 2 weeks later.

Sample and location

Expert panel members will be included from different regions in the world including experts from the United Kingdom, United States, Arab Countries, Africa and from the World Health Organization. The panel members are chosen because of their extensive and invaluable input into the field of patient safety evidenced by their publications in the field. An email will be circulated to each member of the expert panel by the researcher inviting them to participate and respond to the researcher via email to confirm their willingness to participate in the study. Those who wish to participate will be contacted by the researcher with the survey link and a copy of the participant information sheet and consent form. Each questionnaire round will be circulated via email to be completed in the privacy of the individual's home to avoid bias or coercion. All responses within the expert panel will be anonymised prior to collation and circulation.

Analysis

Descriptive statistics will be used to evaluate consensus agreement, including percentage agreement and coefficient of variation. The latter is a normalised measure of dispersion of a probability distribution. The coefficient of variation (CV) is defined as the ratio of the standard deviation to the mean. A level of consensus will also be established. Kendall's coefficient of concordance will be used to evaluate

For peer review only - http://bmjopen.bmj.com/site/about/guidelines.xhtml

consensus across all participants. This is a non-parametric statistic. It can be used for assessing agreement among the participants. Kendall's *W* ranges from 0 (no agreement) to 1 (complete agreement).

The Analysis will include:

- Mean rating
- Percentage agreement
- Coefficient of variation
- Kendall's coefficient of concordance

Consensus will be established if:

- Mean rating of 3.5
- Percentage agreement of ≥ 75%
- Coefficient of variation of ≤ 20%
- A median ranking of 4 or more

DISCUSSION

For patient safety to be truly global, and as highlighted by the Institute of Medicine, low and middle-income countries need to be included in the patient safety community[30]. Thus, it could be argued that over the coming 15 years, harm reductions are likely to come from these countries. More importantly, there is a need to ensure that those tools which have been developed in high-income countries can also be developed in low income countries. Countries in transition such as Libya can also be fertile for innovation in the healthcare sector. There is also a need for the healthcare system in developing countries to have the right capabilities to produce changes in the delivery and design of services so that the quality and value of healthcare in the 21st century can be improved. Satisfaction, biological and functional parameters should be used to measure the outcomes as part of the re-design efforts of healthcare systems[31]. The capacity of the healthcare system should be built for improvement by successful healthcare leaders of the 21st century in developing countries. Policy makers and all stakeholders in healthcare organisations in developing countries should focus on the need of aligning all organisational strategies for improvement (professional development, financing, operations and education) and should perform patient assessment, data gathering, process assessment and outcome measurement to achieve organisational improvement [32]. In other words, the professional environment, staff, patient and family as well as the leadership and management should all be engaged in all phases of the improvement work. For example, the management and leadership of healthcare organisations can play a significant role in improving patient safety through the establishment of caring relationship with clinical staff, building patient safety infrastructure and promotion of organisational culture change in support the quality and patient safety improvement efforts[33 34]. The absence of legal frameworks regarding patient safety improvement along with limited resources and expertise in the field could also influence upon the capabilities of health systems in developing countries. Due to the limited resources, investment in information technology systems in developing countries may not be possible and that will impact upon the overall improvement of care delivered to patients.

An agreed set of indicators that are suitable for international comparison which can also help in forming the basis of local measurement systems is highly needed as part of the global movement for patient safety. Global research on patient safety should shift the focus on investigating the issues that face low-income countries and

For peer review only - http://bmjopen.bmj.com/site/about/guidelines.xhtml

countries in transition in improving their patient safety performance[35]. Research collaboration across countries and international organisations would be very beneficial for countries that have poor infrastructure for conducting empirical research studies. The findings of research could also be disseminated on a global and regional scale to help low-income countries translate these findings into policy recommendations. Patient safety requires a systems approach as it is highly dependent on systemic factors. Redesigning the healthcare system for safer care delivery requires a systems approach in which individual and organisational factors are taken into account[36]. Healthcare institutions would be vulnerable to mistakes in patient safety if a narrow focus on one isolated process without taking into consideration the systematic nature of medical errors is adopted[37]. Hence, this modified Delphi study will help in establishing a point of reference for policy makers in developing countries on the vital elements and components that are required when developing a national strategy for patient safety improvement. This research would also help in bridging the gaps between developed and developing nations and to encourage collaborative research projects between institutions and researchers so that sustainable solutions and improvements can be made.

ETHICS AND DISSEMINATION

Ethical approval was granted by Imperial College Research Ethics Committee (ICREC: 16IC3598). Confidentiality and anonymity will be kept throughout the study phases and all individual responses will be anonymised prior to collation by the researchers involved in the analysis of this study. Only members of the research team will have access to the codes that will be used for coding the Delphi questionnaires. Data which will be published will not identify the identities or organisations of the individuals participated in the study. The findings of the study will be published in a PhD thesis. A manuscript will also be prepared for publication in a high-impact peer-reviewed journal describing the modified Delphi process and the findings of the study. We also expect that the findings of this study will be disseminated to national, international and regional audiences via conference presentations, posters and seminars. Participants in the Delphi study will also be forwarded a copy of the final report. Finally, it is expected that the findings of this study will initiate collaborative research between countries in developing and developed nations about improving patient safety.

CONCLUSION

All healthcare professionals and stakeholders in the process of healthcare delivery should have personal responsibility to patient safety. A positive culture of patient safety is highly needed in developing countries to ensure that discovering and reporting errors is being rewarded not punished. This modified Delphi study will help in identifying the key priorities for the improvement of patient safety in developing countries using Libya as a case-study and will also help shifting the focus of policy makers in these countries on the key important elements that are needed when developing a national strategy for patient safety improvement. The study will identify the barriers that hinder the implementation of patient safety systems so that comprehensive and efficient national patient safety policies and strategies are developed.

Contributorship Statement

For peer review only - http://bmjopen.bmj.com/site/about/guidelines.xhtml

ME and RB prepared the study design and the content of the study protocol. MA further improved the quality of the protocol with important intellectual revisions and assisted in the development of the survey questionnaire for round one. ME is responsible for drafting the protocol manuscript which was then revised and approval by RB and MA.

Competing Interests

None

Funding

This study is part of ME's PhD research which was funded by the Government of Libya. The Department of Primary Care & Public Health at Imperial College London is grateful for support from the NW London NIHR Collaboration for Leadership in Applied Health Research & Care (CLAHRC), the Imperial NIHR Biomedical Research Centre, and the Imperial Centre for Patient Safety and Service Quality (CPSSQ).

Data Sharing Statement

No additional data are available

For peer review only

REFERENCES

1. Vincent C, Amalberti R. *Safer Healthcare*.; 2016. doi:10.1007/978-3-319-25559-0.
2. Landrigan CP, Parry GJ, Bones CB, Hackbarth AD, Goldmann DA, Sharek PJ. Temporal Trends in Rates of Patient Harm Resulting from Medical Care. *N Engl J Med*. 2010;363(22):2124-2134. doi:10.1056/NEJMsa1004404.
3. Vincent C, Burnett S, Carthey J. Safety measurement and monitoring in healthcare: a framework to guide clinical teams and healthcare organisations in maintaining safety. *BMJ Qual Saf*. 2014;23(April):670-677. doi:10.1136/bmjqs-2013-002757.
4. Francis R. *Report of the Mid Staffordshire NHS Foundation Trust Public Inquiry*. Vol 2013.; 2013. doi:10.1002/yd.20044.
5. Wolfe a. Institute of Medicine Report: Crossing the Quality Chasm: A New Health Care System for the 21st Century. *Policy, Polit Nurs Pract*. 2001;2(3):233-235. doi:10.1177/152715440100200312.
6. Carayon P, Schoofs Hundt A, Karsh B-T, et al. Work system design for patient safety: the SEIPS model. *Qual Saf Health Care*. 2006;15 Suppl 1:i50-8. doi:10.1136/qshc.2005.015842.
7. Donabedian a. The quality of medical care. *Science (80-)*. 1978;200(4344):856-864. doi:10.1126/science.417400.
8. Pronovost PJ, Holzmueller CG, Ennen CS, Fox HE. Overview of progress in patient safety. *Am J Obstet Gynecol*. 2011;204(1):5-10. doi:10.1016/j.ajog.2010.11.001.
9. Vincent CA, Coulter A. Patient safety: what about the patient? *Qual Saf Health Care*. 2002;11(1):76-80. doi:10.1136/qhc.11.1.76.
10. Brennan TA, Leape LL, Laird NM, et al. Incidence of adverse events and negligence in hospitalized patients. Results of the Harvard Medical Practice Study I. *N Engl J Med*. 1991;324(6):370-376. doi:10.1056/NEJM199102073240604.
11. Wilson RM, Runciman WB, Gibberd RW, Harrison BT, Newby L, Hamilton JD. The Quality in Australian Health Care Study. *Med J Aust*. 1995;163(9):458-471. http://eutils.ncbi.nlm.nih.gov/entrez/eutils/elink.fcgi?dbfrom=pubmed&id=7476634&retmode=ref&cmd=prlinks%5Cnpapers3://publication/uuid/82A70B92-AB8B-4AD2-A44F-F45DC132B93A.
12. Wilson RM, Michel P, Olsen S, et al. Patient safety in developing countries: retrospective estimation of scale and nature of harm to patients in hospital. *Bmj*. 2012;344(mar13_3):e832. doi:10.1136/bmj.e832.
13. Harrison R, Cohen AWS, Walton M. Patient safety and quality of care in developing countries in Southeast Asia: A systematic literature review. *Int J Qual Heal Care*. 2015;27(4):240-254. doi:10.1093/intqhc/mzv041.
14. Schwappach DLB, Conen D. Patient safety - Who cares? *Swiss Med Wkly*. 2012;142(JULY). doi:10.4414/smw.2012.13634.
15. Carpenter KB, Duevel M a, Lee PW, et al. Measures of patient safety in developing and emerging countries: a review of the literature. *Qual Saf Health Care*. 2010;19(1):48-54. doi:10.1136/qshc.2008.031088.
16. Donaldson LJ, Fletcher MG. The WHO World Alliance for Patient Safety: Towards the years of living less dangerously. *Med J Aust*. 2006;184(10

For peer review only

For peer review only - http://bmjopen.bmj.com/site/about/guidelines.xhtml

SUPPL.). doi:don10047_fm [pii].
17. Hsu C, Sandford B. The delphi technique: making sense of consensus. *Pract Assessment, Res Eval*. 2007;12(10):1-8. doi:10.1016/S0169-2070(99)00018-7.
18. Rowe G, Wright G. The Delphi technique: Past, present, and future prospects - Introduction to the special issue. *Technol Forecast Soc Change*. 2011;78(9):1487-1490. doi:10.1016/j.techfore.2011.09.002.
19. Ziglio E. The Delphi Method and its Contribution to Decision-making. In: *Gazing Into the Oracle: The Delphi Method and Its Application to Social Policy and Public Health*. ; 1996:252.
20. Falzarano M, Pinto Zipp G. Seeking consensus through the use of the Delphi technique in health sciences research. *J Allied Health*. 2013;42(2):99-105.
21. Landeta J. Current validity of the Delphi method in social sciences. *Technol Forecast Soc Change*. 2006;73(5):467-482. doi:10.1016/j.techfore.2005.09.002.
22. Thomson R, Lewalle P, Sherman H, Hibbert P, Runciman W, Castro G. Towards an International Classification for Patient Safety: A Delphi survey. *Int J Qual Heal Care*. 2009;21(1):9-17. doi:10.1093/intqhc/mzn055.
23. Verstappen W, Gaal S, Esmail A, Wensing M. Patient safety improvement programmes for primary care. Review of a Delphi procedure and pilot studies by the LINNEAUS collaboration on patient safety in primary care. *Eur J Gen Pract*. 2015;21 Suppl:50-55. doi:10.3109/13814788.2015.1043725 [doi].
24. Elwyn G, O'Connor A, Stacey D, et al. Developing a quality criteria framework for patient decision aids: online international Delphi consensus process. *BMJ*. 2006;333(7565):417. doi:bmj.38926.629329.AE [pii] 10.1136/bmj.38926.629329.AE.
25. Akins R, Cole BR. Barriers to implementation of patient safety systems in healthcare institutions: leadership and policy implications. *J Patient Saf*. 2005;1(1):9-16. http://journals.lww.com/journalpatientsafety/Abstract/2005/03000/Barriers_to_Implementation_of_Patient_Safety.5.aspx.
26. Barton AJ, Armstrong G, Preheim G, Gelmon SB, Andrus LC. A national Delphi to determine developmental progression of quality and safety competencies in nursing education. *Nurs Outlook*. 2009;57(6):313-322. doi:10.1016/j.outlook.2009.08.003.
27. Haller G, Camparini-Righini N, Kern C, et al. [Patient safety indicators for obstetrics: a Delphi based study]. *J Gynecol Obstet Biol Reprod (Paris)*. 2010;39(5):371-378. doi:10.1016/j.jgyn.2010.05.011.
28. Farley DO, Greenberg MD, Haviland AM, Lovejoy S. Prioritizing Patient Safety Outcomes Measures: Results of an Expert Consensus Process. *Rand Heal*. 2008.
29. Nigam R, Mackinnon NJ, U D, et al. Development of canadian safety indicators for medication use. *Healthc Q*. 2008;11(3 Spec No.):47-53. doi:10.12927/hcq.2008.19649.
30. Zhu H, Das P, Woodhouse R, Kubba H. Improving the quality of tracheostomy care. *Breathe*. 2014;10(4):286-294. doi:10.1183/20734735.011114.
31. Batalden P, Splaine M. What will it take to lead the continual improvement and innovation of health care in the twenty-first century? *Qual Manag Health Care*. 2002;11(1):45-54. doi:10.1097/00019514-200211010-00008.
32. Brandrud AS, Schreiner A, Hjortdahl P, Helljesen GS, Nyen B, Nelson EC. Three success factors for continual improvement in healthcare: an analysis of

For peer review only

For peer review only - http://bmjopen.bmj.com/site/about/guidelines.xhtml

the reports of improvement team members. *BMJ Qual Saf*. 2011;20:251-259. doi:10.1136/bmjqs.2009.038604.

33. Bradley EH, Holmboe ES, Wang Y, et al. What are hospitals doing to increase beta-blocker use?. *Jt Comm J Qual Saf*. 2003;29(8):409-415. http://ovidsp.ovid.com/ovidweb.cgi?T=JS&PAGE=reference&D=med4&NEWS=N&AN=12953605.
34. Künzle B, Kolbe M, Grote G. Ensuring patient safety through effective leadership behaviour: A literature review. *Saf Sci*. 2010;48(1):1-17. doi:10.1016/j.ssci.2009.06.004.
35. Bates DW, Larizgoitia I, Prasopa-Plaizier N, Jha AK. Global priorities for patient safety research. *BMJ*. 2009;338(may):b1775. doi:10.1136/bmj.b1775.
36. Waterson P. A critical review of the systems approach within patient safety research. *Ergonomics*. 2009;52(10):1185-1195. doi:10.1080/00140130903042782.
37. McNutt RA, Abrams R, Arons DC. <McNutt Patient safety efforts should focus on medical errors.pdf>. *JAMA*. 2002;287(15):1997-2001. doi:10.1001/jama.287.15.1997.

For peer review only

For peer review only - http://bmjopen.bmj.com/site/about/guidelines.xhtml

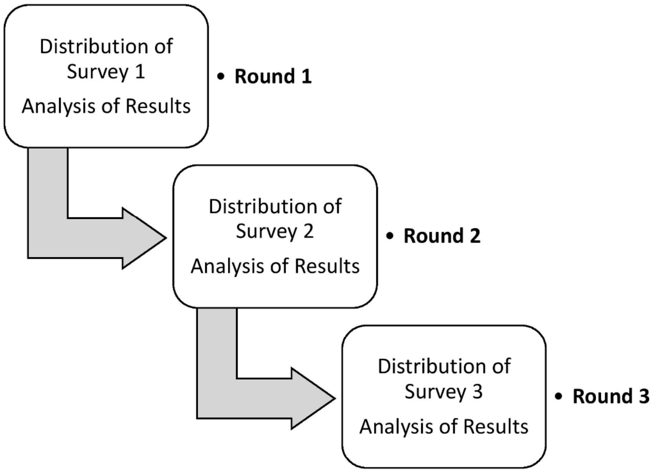

Figure 1

210x297mm (300 x 300 DPI)

Mustafa Elmontsri
PhD Student
Department of Primary Care and Public Health
Imperial College London

Room 309, Reynolds Building
St Dunstan's Road
London W6 8RP
Tel: +44 (0)7716048560

m.elmontsri10@imperial.ac.uk
www.imperial.ac.uk/medicine/people/m.elmontsri10

Title of Project: Development and Validation of a Patient Safety Improvement Model for Libyan Healthcare Settings

Delphi Questionnaire for Round One

Key priority areas for patient safety improvement in developing countries (Libya as case study): a modified Delphi study

Instructions
The following table presents suggested items, areas to address, critical processes and for patient safety improvement in healthcare institutions in developing countries as identified in current literature on patient safety in healthcare and by national patient safety organisations such as the World Health Organization.

When ranking the elements, please remember that these elements are aimed at improving patient safety in Libya which is witnessing a major challenges at the moment as a consequence of the war. In other words, to what extent do you believe that each element is of a high importance at this stage to help healthcare institutions in Libya provide a relatively safer care to patients?

Please place a rank in each respective "Rank" column for each element under each domain. Rank each element from 1 to 5, in the context of its importance to improving patient safety in developing countries, where;

- "5" represents "very important"
- "4" represents "important"
- "3" represents "no opinion"
- "2" represents "not very important"
- "1" represents "unimportant"

Please also give an overall rank of importance to each domain from 1 to 5.

Thank you for your time and effort for filling in this questionnaire

For peer review only - http://bmjopen.bmj.com/site/about/guidelines.xhtml

Domain	Element	Rank current importance	Overall Domain importance rank
**1. REGULATION AND GOVERNANCE**	**1.1** Introducing patient safety law at parliament level		
	**1.2** Establishing an independent patient safety agency in Libya to regulate healthcare providers		
	**1.3** Introducing evidence-based Patient Safety Indicators for all healthcare institutions		
	**1.4** Setting standards for the government bodies that regular healthcare providers, professions and specialist areas of medicine		
**Other (please state)**			
**Domain**	**Item**	**Rank current importance**	**Overall Domain importance rank**
**2. LEADERSHIP AND MANAGEMENT**	**2.1** Senior leaders communicate the priority of patient safety to all stakeholders.		
	**2.2** Ensuring that procedures are developed and approved by the governing body when required		
	**2.3** Leaders and managers should incorporates patient safety accreditation and legal requirements as integral parts of its performance improvement.		
	**2.4** Leaders and managers should develop plans and support systems for proactive collecting and analysis of patient safety information for future improvement		
**Other (please state)**			
**Domain**	**Item**	**Rank current importance**	**Overall Domain importance rank**
**3. INFORMATION TECHNOLOGY**	**3.1** Introducing Electronic Medical Records for managing patient's information		
	**3.2** Using technology for the prescribing of medications		
	**3.3** Providing opportunities for patient to access their medical records		
	**3.4** Providing training for all healthcare staff in using IT		
**Other (please state)**			
**Domain**	**Item**	**Rank current importance**	**Overall Domain importance rank**

For peer review only - http://bmjopen.bmj.com/site/about/guidelines.xhtml

**4. EDUCATION AND TRAINING**	**4.1** Ensuring that there is a staff professional development programme with patient safety as a cutting theme		
	**4.2** Embedding patient safety in all levels of education and training for healthcare professionals (i.e. undergraduate and postgraduate training, continuous improvement		
	**4.3** Ensuring that training curricula are high-quality, locally and clinically relevant, and delivered effectively.		
	**4.4** Developing mechanisms for periodic gathering of information on healthcare providers' opinions and expectations in regard to factors		
**Other (please state)**			
**Domain**	**Item**	**Rank current importance**	**Overall Domain importance rank**
**5. MEASUREMENT, ANALYSIS AND MOINTORING**	**5.1** Introducing Incident Reporting System for all healthcare institutions		
	**5.2** Ensuring that data collected from reporting systems are used for future learning to improve service delivery		
	**5.3** Measuring patient safety culture among healthcare staff at all levels		
	**5.4** Ensuring that Clinical performance measures as developed by national, regional or professional institutions (as applicable) are implemented in the everyday clinical practice		
**Other (please state)**			
**Domain**	**Item**	**Rank current importance**	**Overall Domain importance rank**
**6. PATIENT AND PUBLIC INVOLVEMENT**	**6.1** Implementing a patient satisfaction questionnaire.		
	**6.2** Providing training in patient satisfaction and interpersonal communication for staff		
	**6.3** Ensuring that written policies on patient's rights are available, disseminated, or made visible to patients and their families		
	**6.4** Ensuring that patients are encouraged to speak up and act upon the patient's voice		
**Other (please state)**			

For peer review only - http://bmjopen.bmj.com/site/about/guidelines.xhtml

Domain	Item	Rank current importance	Overall Domain importance rank
**7. INFRASTRUCTURE AND WORKING ENVIRONMENT**	**7.1** Staff and patients are protected from health and safety hazards (noise, display screen equipment, manual handling etc...)		
	**7.2** Ensuring that a system to reduce risk of health care-associated infections is implemented		
	**7.3** Developing a safe waste management system according to international standards		
	**7.4** Developing and enforcing **I**nfection control policies (hand washing, injection waste, blood transfer etc...)		
**Other (please state)**			

Please list the top five barriers to implementation of patient safety systems in healthcare organisations in developing countries:

1. ....
2. ....
3. ....
4. ....
5. ....

For peer review only - http://bmjopen.bmj.com/site/about/guidelines.xhtml
